# Drug-loaded nanoparticles for intra-articular injection

**DOI:** 10.1371/journal.pone.0327958

**Published:** 2026-01-16

**Authors:** Piaopiao Pan, Konstantina Simou, Yanting Ouyang, Lawrence Shere, Jon A. Preece, Simon W. Jones, Edward T. Davis, Yanling Lan, Zhenqiu Chen, Zhenyu Jason Zhang, Qingguo Li

**Affiliations:** 1 School of Pharmaceutical Sciences, Guangzhou University of Chinese Medicine, Guangzhou, PR China; 2 Centre for the Microenvironment, School of Molecular Bioscience, University of Glasgow, Glasgow, United Kingdom,; 3 School of Chemical Engineering, University of Birmingham, Birmingham, United Kingdom; 4 Guangzhou University of Traditional Chinese Medicine ShunDe Traditional Chinese Medicine Hospital, Foshan, Guangdong, PR China; 5 School of Chemistry, University of Birmingham, Birmingham, United Kingdom; 6 Institute of Inflammation and Ageing, University of Birmingham, Birmingham, United Kingdom; 7 The Royal Orthopaedic Hospital, NHS Foundation Trust, Birmingham, United Kingdom; 8 The First Affiliated Hospital, Guangzhou University of Chinese Medicine, Guangzhou, PR China; University of South Carolina, UNITED STATES OF AMERICA

## Abstract

Drug loaded nanoparticles (NPs) were developed as a model intra-articular injection (IAI) formulation to mitigate early stage osteoarthritis (OA). Different types of celecoxib-loaded nanoparticles were prepared by a hybrid method that combines homogenization and solvent evaporation. The hydrodynamic diameter of the nanoparticles prepared were approximately 200 nm (PLLA: 238 ± 19 nm; PCL: 249 ± 28 nm; PLA: 252 ± 18 nm; PMMA: 234 ± 21), and zeta potential were about −40 mV (PLLA: −45.3 ± 2.3 mV; PCL: −38.0 ± 0.9 mV; PLA: −44.4 ± 3.2 mV; PMMA: −45.5 ± 2.7 mV). Our friction data evidences that nanoparticles could improve considerably the lubrication between a stainless steel sphere and a silicone elastomer that were used as model substrates. Quartz Crystal Microbalance (QCM) and Atomic Force Microscope (AFM) measurements were carried out to unravel the lubrication mechanism. The magnitude and amount of NPs adsorbed on the surface determines the effect of lubrication. Drug release experiment suggests that nanoparticles could release up to more than one week, when being compared with free celecoxib. NPs formulation exhibited excellent biocompatibility in cytotoxicity of chondrocytes experiment.

## Introduction

Osteoarthritis (OA), known as “wear and tear” arthritis, is a joint disease that is triggered by the degeneration of articular cartilage and joint inflammation, resulting in lubrication deficiency, which accelerates the deterioration. It affects millions of people worldwide, and is cited as the second most common reason for time requested off work [1]. Direct costs related to OA amounted to>£1Bn in 2010 in UK [2], over $60 billion in 2007 in US, and the aggregate cost of OA is expected to increase to $185.5 billion per year based on data from 2007 [3]. The primary strategies to mitigate OA, including surgical, non-pharmacological, and pharmacological treatments, are to reduce pain and improve joint mobility. It is common to delay surgical intervention for several years by non-pharmacological and pharmacological treatments because of the high cost and low acceptance of patients. Non-pharmacological treatments include weight loss, exercise, and knee bracing that mechanically stabilize the joint [4,5]. Pharmacological treatment includes administration of non-steroidal anti-inflammatory drugs (NSAIDs), COX II inhibitors, and oral non-narcotic analgesics [6], none of which provides a satisfactory result since there is no blood circulation in a cartilage [7]. The technical barrier to deliver a selective therapy systematically makes effective treatment for OA more difficult.

Articular cartilage is a porous matrix, of which the function is determined by the molecular composition and structural characteristics of its extracellular matrix (ECM) [8]. As OA progresses, cartilage gradually loses its exceptional lubrication properties and the viscosity of synovial fluid changes [9]. To compensate for the reducing tribological performance of the articular joint, the concept of Intra-Articular Injection (IAI) of a viscosupplementation containing hyaluronic acid (HA) was introduced. The increased concentration of HA in synovial fluid could increase viscosity and reduce inflammation by limiting the activation of interleukin-1 [10]. The effectiveness of viscosupplementation for mild to moderate arthritis and steroid or hyaluronate injections for severe arthritis has been demonstrated in the past [10,11].

Despite the possible benefits of HA-based viscosupplementation, clinical evidence of its effectiveness and long term performance were inconsistent. It was reported that HA is incapable of load-bearing required by the joint, hence increasing the HA concentration in synovial fluid does not necessarily mitigate the adverse impact of OA [12]. In viscosity measurements [13], it was shown that HA does not adsorb onto mica surfaces, and damage was incurred to the surface as a result of shear stress, which suggests that HA is unable to act as a boundary lubricant. Additionally, local HA administration could be cleared rapidly due to synovial fluid exchange. In a degradation experiment of hyaluronidase [14], the lubrication effect of HA was negated, and only participants of joint lubrication were recognized, which suggests that HA molecules do not necessarily stay in the synovial cavity. Its porous nature allows the transport and migration of anti-inflammatory drug molecules to diffuse away, which makes the intra-articular injection a challenging approach to treat rheumatoid arthritis and osteoarthritis.

In recent years, the development of polymer-based nanomedicines for arthritis [15–23] therapy has been reported, whereby studies have demonstrated that nanoparticles play an important role in fields varying from lubrication [24,25] to drug delivery systems. [26,27] From the perspective of joint lubrication, nanoparticles act as interfacial additives to reduce friction between cartilage or its surrogate. The role of nanoparticle concentration, size, shape, and structure were comprehensively reviewed [28]. The effects of the mechanical properties of nanoparticles as lubricant additives on the tribological properties differ in various materials. For polymer nanoparticle, the surface interaction and adsorption of nanoparticles play a critical role in reducing interface friction [29,30]. Apart from lubrication, numerous applications of nanoparticles in the field of drug delivery system have been reported in the past three decades to achieve long-term drug release. Some drug-loaded nanoparticles were investigated as additives to treat OA. For example, Morgen *et al.* [31] demonstrated the feasibility of using cationic polymeric nanoparticles composed of poly (caprolactone) (PCL) and poly (ethylene oxide) (PEO) diblock copolymer crosslinked with anionic HA (dextran) for OA therapy. After intra-articular injections in rat knees, 70% of nanoparticles were retained in the joint for 1 week. Another study reported the delivery vehicle for cationic peptides [32], whereby formulated PEGylated pNiPAM nanoparticles with degradable disulfide crosslinkers were used to deliver anti-inflammatory peptides into chondrocytes. The results of this study revealed a passive targeting of inflamed cartilage *ex vivo* and a suppression of inflammation in various cell types. In addition, recent studies highlight promising strategies to treat bone disorders by focusing on targeted delivery and regulation of key signaling pathways. MiR-665 can promote bone formation by inhibiting sclerostin and activate Wnt signaling [33], while plant-derived nanovesicles from Rhizoma Drynariae enhance Osteogenic differentiation through naringin and estrogen receptor-α [34]. Additionally, localized delivery, such as topical patches, has demonstrated effective symptom relief in Osteoarthritis with reduced side effects [35]. These findings underscore the potential of novel, targeted approaches for improving bone health and joint function.

To date, relatively few studies have successfully integrated the lubricating properties of nanoparticles with their drug delivery functions, particularly in the context of intra-articular interventions for osteoarthritis. A set of viscosupplementation formulation that contains drug-loaded nanoparticles using polymeric matrix were designed in our work. Both tribological behavior and drug release of the nanoparticles were investigated. An intra-articular injection of such drug-loaded nanoparticles into the joint can achieve both lubrication improvement during joint movement and sustained drug release via local administration. This hypothesis is backed by previous, similar experiments [36,37]. Yan and colleagues prepared poly (3-sulfopropyl methacrylate potassium salt)-grafted mesoporous silica nanoparticles inspired by Euryale Ferox seed to treat OA [36]. Although the results revealed that improvement has been achieved by nanoparticles, the drug release was limited due to the nanocarrier and the complex methods of synthesis. Particularly, mesoporous silica is not biodegradable.

**Fig 1:**
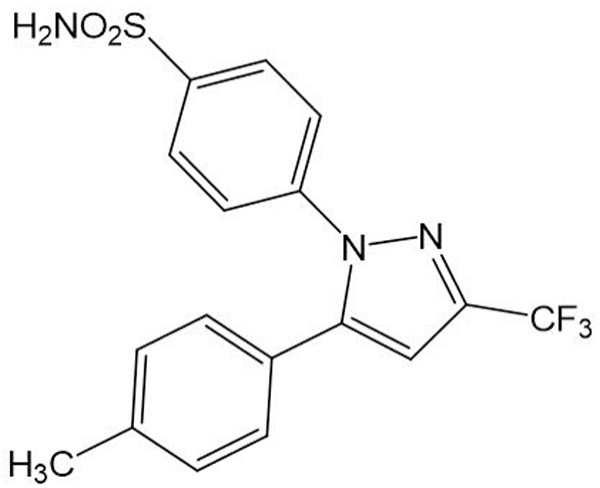
Molecular structure of celecoxib.

In the present study, celecoxib (Fig 1) was chosen as a model drug due to its outstanding anti-inflammatory effect: it is the first COX-2 specific inhibitor approved for use in patients with osteoarthritis and rheumatoid arthritis [38]. Celecoxib has a p*K*a of 11.1, and its low aqueous solubility (∼5 μg mL-1) contributes to a low oral bioavailability ranging from 22%−40%after oral administration [39]. However, it was reported that celecoxib has gastrointestinal stimulation and could not reach the target as there is no blood circulation to cartilage [40,41]. Some formulations were investigated to treat OA by intra-articular injection using celecoxib. For example, Petit *et al.* [42] prepared acetyl-capped PCLA-PEG-PCLA triblock copolymers gels containing celecoxib to treat the joint of horse. The results of Jiang suggest [43] that intra-articular injection of celecoxib is an effective therapeutic method in an OA model. The main source of pain caused by joint friction could not be mitigated despite the anti-inflammatory effect of celecoxib. Celecoxib might not only provide substantially benefits to OA by suppressing of the levels of TNF-α and IL-1, but also maintain cellular activity and extracellular matrix synthesis by reducing MMP-3 synthesis [43]. To achieve the dual function desired for viscosupplementation formulation, a prolonged drug release profile is required.

Polylactic acid (PLA), poly (L-lactide) acid (PLLA), polycaprolactone (PCL), and polymethyl methacrylate (PMMA) were used as nanocarrier in this study. These polymers are biocompatible and biodegradable, except PMMA [27,44]. A number of studies investigated their safety and sustained release effect. Many examples of the potential benefit using nanoparticles in the treatment of various diseases have been demonstrated as a therapeutic strategy [45–48]. The focus of the present study was on the dual benefits of nanoparticle based IAI formulations as an effective intervention to treat early stage OA, whereby the biocompatible nanoparticles could not only provide a selective and prolonged drug release profile, but an improved lubrication at the joint contact interface. Meanwhile, the lubrication mechanism was investigated: a particular focus was given to the tribological behaviors of celecoxib-loaded nanoparticles, including MTM, QCM, and AFM measurements, demonstrating the effect and mechanism of lubrication. Furthermore, drug release and cytotoxicity experiments were carried out to investigate the sustained release and biocompatibility of nanoparticles.

## Results and discussion

### Characteristics of celecoxib loaded nanoparticles

A range of celecoxib loaded polymeric nanoparticles, including PLA, PLLA, PCL, and PMMA, were prepared by high pressure homogenization, in combination with solvent evaporation, which is one of the most common method used for the preparation of nanoparticles [49,50]. Characteristics of the prepared nanoparticles are presented in Table 1.

**Table 1:** Characteristics of celecoxib loaded nanoparticles.

Sample	Initial		After lyophilized		Zeta potential [mV]	Drug Loading [%]	Drug realease at 24 h [%]	Drug realease at 72 h [%]
	Size [nm]	PDI	Size [nm]	PDI				
**PLLA**	238 ± 19	0.13 ± 0.05	240 ± 33	0.18 ± 0.04	−45.3 ± 2.3	6.63 ± 4.61	67.19 ± 5.67	84.42 ± 5.43
**PCL**	249 ± 28	0.11 ± 0.04	441 ± 87	0.16 ± 0.05	−38.0 ± 0.9	6.45 ± 3.72	78.33 ± 7.30	79.85 ± 6.19
**PLA**	252 ± 18	0.14 ± 0.03	259 ± 27	0.21 ± 0.03	−44.4 ± 3.2	4.07 ± 1.14	80.18 ± 7.89	83.47 ± 7.88
**PMMA**	234 ± 21	0.11 ± 0.03	274 ± 34	0.18 ± 0.06	−45.5 ± 2.7	4.96 ± 2.04	34.59 ± 2.34	41.31 ± 2.47

To eliminate the influence of particle size on the lubrication performance of the formulation, polymeric particles of approximately 240 nm were prepared, as confirmed by the results of dynamic light scattering. Polydispersity Index (PDI) for all suspensions measured is within acceptable range. Stability of these particles were also evaluated by zeta potential. Zeta potential of all nanoparticles, dependent on their surface charge, were found in the region near −40 Mv [51], although PCL particles are slightly less charged, comparing to the other three samples. We also found that there was no notable difference in particle size after nanoparticles were lyophilized except PCL particles that showed a 77% increase.

Our findings are in line with prior studies that have utilized polymeric nanoparticles for sustained drug delivery and cartilage targeting. For instance, Martina et al. developed dexamethasone-loaded PLGA nanoparticles for intra-articular injection and reported prolonged drug retention and reduced synovial inflammation in OA models [52]. Similarly, Wen et al. encapsulated curcumin in chitosan-based nanoparticles to enhance its solubility and anti-inflammatory efficacy [53]. Compared to these systems, our celecoxib-loaded NPs exhibited comparable size (∼200–250 nm) and surface charge (~–40 mV), which are known to enhance synovial retention by avoiding rapid lymphatic clearance.

### Friction results

**Fig 2:**
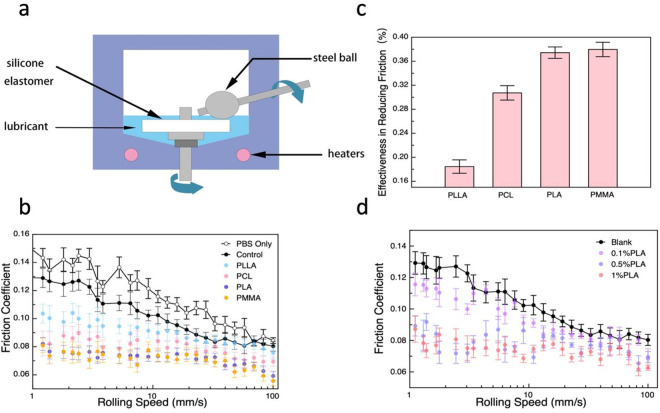
Results of Friction tests. a) Diagram of the MTM. b) Friction coefficient of nanoparticles tested by MTM on steel ball to silicone elastomer set-ups, the concentration of nanoparticles were 0.5% PBS containing 0.5% SDS and 0.1% HA were taken as control. c) Effectiveness in reducing friction tested by MTM on steel ball to silicone elastomer set-ups when rolling speed was below 5 mm/s, compared to the blank group, PBS containing 0.5% SDS and 0.1% HA were taken as control. d) Friction coefficient of different concentration of PLA tested by MTM on steel ball to silicone elastomer set-ups, compared to the blank group which was PBS containing 0.5% SDS and 0.1% HA.

Upon the successful preparation of polymeric nanoparticle loaded with celecoxib, lubrication performance was evaluated using a Mini-Traction-Machine (MTM) over a range of sliding velocity (1−100 mm s^-1^). Tribological characteristics was measured at 37^o^C between a stainless-steel sphere of 19.05 mm diameter and a silicone elastomer substrate, both of which were submerged in the nanoparticle-containing formulation that contains the polymeric nanoparticles, hyaluronic acid, and surfactant, as demonstrated in a previous work (Fig 2a) [54]. Coefficients of Friction (CoF), acquired by the function of sliding velocity. Phosphate Buffered Saline (PBS) containing 0.5% SDS and 0.1% HA was used as control/blank for all measurements.

Fig 2b shows representative Stribeck curves that capture the tribological characteristics of two solid surfaces sliding against each other. It is not surprising that the CoF decreases with an increasing velocity, as we have demonstrated in a previous study [54]. The CoF acquired between stainless steel and silicone elastomer is also consistent with values reported in the literature [55]. Upon the introduction of nanoparticles (0.5% w/v), a significant reduction in terms of CoF was observed for all four types of nanoparticle suspension throughout the range of velocity surveyed (Fig 2c): the friction was reduced by up to 38% through introducing PMMA nanoparticles.

Effect of nanoparticle concentration on the frictional properties was also evaluated in the present work, using PLA nanoparticles of 0.1%, 0.5%, and 1%. Fig 2d suggests that increasing the concentration from 0.1% to 0.5% had a significant impact on the macroscopic friction, in both the values of CoF and the nature of the contact mechanics, which is consistent to our previous study whereby blank nanoparticles (identical polymeric matrix, no celecoxib) were used. It is very probable that the surface deposited nanoparticles could improve the smoothness of the silicone elastomer, which reduces the asperities in contact, and subsequently decrease friction. However, no noticeable difference was observed when PLA nanoparticle concentration was increased from 0.5% to 1%, which implies that the surface coverage of nanoparticles was adequate at 0.5%. Both the distinctive different between 0.1% and 0.5% concentration, and the insignificant variation between 0.5% and 1% highlight the critical role of surface deposited nanoparticles in lubricating an articulating interface that replicates cartilage at the early stage osteoarthritis.

### Surface adsorption of nanoparticles

To validate the hypothesis that surface adsorption of nanoparticles plays a critical role, and to evaluate quantitatively the corresponding kinetics, a quartz crystal microbalance (QCM) was deployed. A self assembled monolayer (SAM) of (3-Mercaptopropyl) trimethoxysilane (MPTMS) [56] was prepared on a gold-coated QCM crystal to replicate the chemical nature of the silicone elastomer used in the frictional tests. Water contact angle and thickness of the formed monolayer were 66 ± 1° and 0.3 ± 0.1 nm, respectively. The fresh SAM is slightly hydrophilic as expected from the presence of methoxy groups. The elipsometric thickness of the SAM is 0.28 ± 0.11 nm, which is shorter than the expected calculated thickness of 0.7 nm of a fully elongated molecule in the all trans conformation of bonds [57]. However, SAMs on the gold substrate are disordered, which coupled to the short length of the molecule will undoubtedly mean that the molecules in the SAM are not in the all trans extended conformation, and not all surface silica sites will be filled, leading to a suboptimally covered and ordered SAM and lead to the lower than expected thickness [58,59].

**Fig 3:**
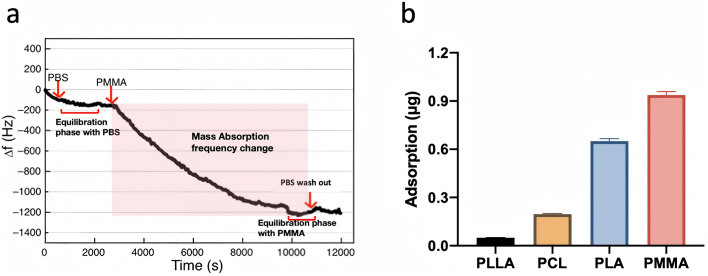
Adsorption of nanoparticles on SAM. a) Measurement of representative nanoparticles (PMMA nanoparticles in this case) adsorbed on silane SAMs by QCM. b) Adsorption of different nanoparticles on SAM-coated crystal measured by QCM.

Fig 3a shows a representative adsorption measurement whereby a MPTMS functionalized QCM sensor was exposed to a PBS buffer solution until the frequency reaches an equilibrium when in contact with de-ionised water (no change with frequency), and subsequently to a suspension containing 0.1% nanoparticles of interest. Surface adsorption of the nanoparticles resulted in a reduction in the frequency, which was monitored in real time as changes with frequency of the sensor. The QCM chamber was then rinsed with PBS solution to evaluate the magnitude of surface attachment of the nanoparticles.

The total mass of the adsorbed nanoparticles can be calculated from the frequency change according to Sauerbrey equation (1).


$$\Delta \text{f}=-\frac{\text{2}f_{\text{0}}^{\text{2}}}{A\sqrt{\rho_{q}\mu_{q}}}\Delta m$$ (1)

where *f*_0_ is the resonant frequency of crystal (Hz), ∆*f* is frequency change (Hz), ∆*m* is mass change (g), *A* is piezoelectrically active crystal area (Area between electrodes, cm^2^), *ρ*_q_ is density of quartz (*ρ*_q_ = 2.648 g/cm^3^) and *μ*_q_ is shear modulus of quartz for AT-cut crystal (*μ*_q_ = 2.947x10^11^ g·cm^-1^·s^-2^).

For an AT cut quartz crystal, the equation can be simplified as:


Δm=A×C×Δf (2)

where C is a constant, of which the crystal used the present work is 4.42 x10^-9^ g Hz^-1^ cm^-2^. All frequency changes, measured as ∆*f* (Hz), were recorded when the dynamic adsorption/desorption process had reached the equilibrium conditions. The active area for adsorption measurement, based on the physical geometry of the QCM sensor, is 0.2043 cm^2^. The adsorption amount of four types of celecoxib-loaded nanoparticles on silane monolayer are shown in Fig 3b. It can be seen that the celecoxib-loaded PMMA nanoparticles produce the most significant adsorption amount, followed by PLA, PCL and PLLA, which follows the same order observed by the friction results. Considering the lubrication mechanism established in our previous tribological work whereby blank nanoparticles were used,^46^ the QCM results confirm that surface adsorption of nanoparticles is very likely a crucial factor introducing lubrication to an articulating interface.

**Fig 4:**
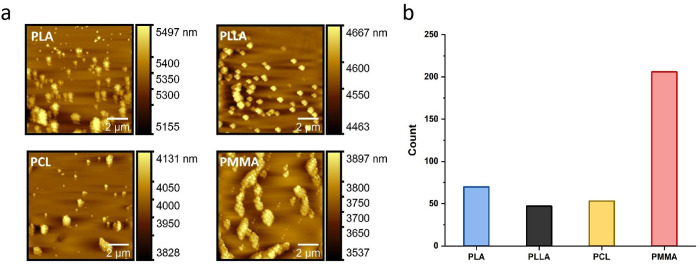
Four types of celecoxib-loaded PLA, PLLA, PCL and PMMA were investigated by AFM. a) AFM images of SAM-coated QCM sensors. The SAM-coated QCM sensors were investigated by AFM after QCM tests in ambient condition. b) Nanoparticle quantification of AFM images in (a) by Image J.

To further confirm the adsorption data acquired by QCM measurements, the SAM-coated QCM sensors were evaluated by AFM after QCM tests in ambient condition. Representative images of celecoxib-loaded PLA, PLLA, PCL and PMMA, are presented in Fig 4. Unlikely our DLS data, suggesting that the nanoparticles were in a dispersed form, the majority of the nanoparticles were found in an aggregate form, which is likely driven by the evaporation of PBS buffer prior to the AFM measurements. The most useful information from this morphological study is the confirmation that the amount of PMMA and PLA adsorbed on the surface is greater than PCL and PLLA, which is consistent with the QCM results. It, once again, highlights that the magnitude of interaction between nanoparticles and silicone elastomer vary, and it could underpin the design of nanoparticle based viscosupplementation formulation for maximized lubrication properties. While most previous formulations have focused solely on drug delivery, our study demonstrates that nanoparticles can also act as boundary lubricants, reducing friction between model joint surfaces. AFM and QCM measurements provided mechanistic insights, revealing that the magnitude and density of NP adsorption on surfaces directly impact their lubricating function. These results align with the lubrication behavior observed in hyaluronic acid-modified liposomes, reported by Lin’s team [60], which also showed friction reduction via surface adsorption mechanisms.

### Drug release

**Fig 5:**
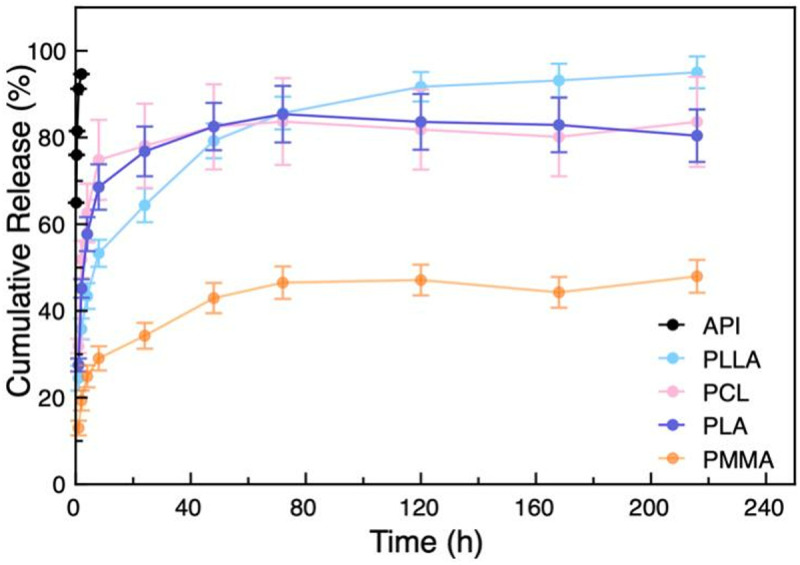
Release profiles of the celecoxib from the celecoxib loaded nanoparticles in PBS buffer solutions under physiological temperature (37°C), including free celecoxib as a control.

Fig 5 presents the release profiles of the drug-loaded nanoparticles, including pure celecoxib as a benchmark. It was found that the accumulated amount of celecoxib without a carrier rapidly reached 100% within the first 4 h. As a comparison, celecoxib-loaded nanoparticles exhibited a significantly improved release behavior over a course of 220 h. PLLA, PCL and PLA release curve are relatively similar, which is faster than PMMA. When PLLA, PCL and PLA were used as the nanocarrier, celecoxib released more than 60% at first 24 h and 80% at 72h, and the release equilibrium was subsequently reached. Celecoxib was incorporated into the polymer matrix, and its release is driven by polymer degradation and drug diffusion. Drug carriers composed of these biodegradable polymers release the drug through hydrolytic and/or enzymatic degradation of ester, amide, and hydrazone bonds in their backbones [61,62]. However, When PMMA was used as the nanocarrier, celecoxib released only 30% at 24h, and then the release gradually slowed down, cumulative released reaching nearly 50% at 220 hours. This may be due to the non-biodegradable and hydrophobicity of PMMA [63]. Although PMMA shows a prolonged release properties than biodegradable polymers PLLA, PLA and PCL, they are significantly longer than what has been reported in the literature. Liu and colleague [64] prepared brushes-grafted hollow silica nanoparticles as a promising platform for joint lubrication, whereby the sustained release lasted up to 70 h. In another study where knee was injected with chitosan microspheres loaded with brucine, the release profile of brucine showed nearly 120 h sustained release [65]. *In vitro* release of celecoxib from the liposome formulation and chitosan microspheres was evaluated for the treatment of osteoarthritis [66,67]. The results of drug release were about 72 h and 96 h, respectively. As comparison, the polymeric nanoparticles prepared in the present work show a prolonged release up to 220 h. Meanwhile, organic polymers provide a safe and effective way to work as a drug carrier. PMMA, a biomaterial that has long been used for biomedical applications, has a good characterization as a biocompatible material which does not usually trigger any immune response from the host [68,69]. This provides new concept for the preparation of safe and prolonged release polymeric nanoparticles.

### Cytotoxicity of nanoparticles suspensions

**Fig 6:**
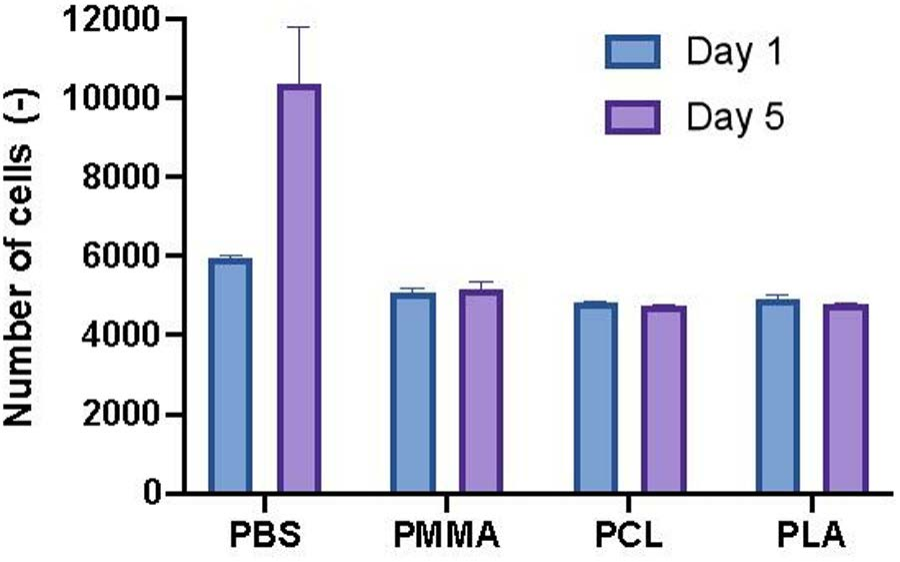
Cytotoxicity of chondrocytes after incubation with different celecoxib loaded nanoparticles. Cell numbers of chondrocytes were evaluated by Microtubules (MTS) array on day 1 (blue) and day 5 (purple). The formulation consisted of (0.5%)/HA, (0.1%)/SDS (0.5%) in PBS. The data was presented as average, the error bars represent the standard error from three wells seeded with the same sample.

The toxicity of the NPs formulations was evaluated on primary human osteoarthritic synovial chondrocytes (Fig 6) The NPs formulation exhibited excellent biocompatibility. After one day of incubation, a reduction of 13–18% on the number of the cells was observed, and there was no additional reduction with time (reduction after five days 11–18%). PMMA formulations were proved the most biocompatible, providing average cell viability of 88%, whereas PLA and PCL provided viability of 83% and 82%, respectively. PMMA suspensions was proved the least toxic from the three NPs, providing a 90.5% cell viability. These results support the potential of this nanoparticle suspension for future in vivo validation and possible clinical translation in OA treatment.

## Conclusion

In the present work, we developed a dual functional nanoparticle-based formulation with great potential for Intra-Articular Injection to treat early stage osteoarthritis. With significantly reduced Coefficients of Friction, celecoxib-loaded nanoparticles show great effect in lubricating the model substrates, driven by surface adsorption of nanoparticles. We highlight that the nature of surface adhesion of NPs could be the key enabler as it ensures the presence of NPs at the contact area. Meanwhile, celecoxib-loaded nanoparticles worked as a sustained formulation in the joint, which far exceeds the release profile of the free drug molecules. IAI of celecoxib-loaded particles remarkably improves the lubrication and sustained drug release on the targeted site, compared to the conventional approaches for early stage of osteoarthritis.

## Materials and methods

### Materials

Polylactic acid (PLA, Shandong Academy of Pharmaceutical Sciences, 0.34 dL/g, LOT: 17011303, *M*_w_ ~ 3−50000), Poly (L-lactide) acid (PLLA, Shandong Academy of Pharmaceutical Sciences, 0.35 dL/g, LOT: 170909011, *M*_w_ ~ 3−50000), Polycaprolactone (PCL, Shyuanye, China, CAS^#^24980-41-4, LOT: Y25A9F59704, *M*_w_ ~ 80000), Polymethyl methacrylate (PMMA, Macklin Biochemical, Shanghai, China, CAS^#^9011-14-7, LOT: C10102099), celecoxib (Boyuan Chemical Co.,Ltd, Shangdong, China), Polyvinyl Alcohol (PVA, Aladdin, *M*_w_ ~ 145000), hyaluronic acid (HA, Bloomage Freda Biopharm Co.,Ltd, *M*_w_ ~ 400000), and sodium docecyl sulfate (SDS, Fisher Scientific, USA) were used as purchased.

### Preparation of drug-loaded nanoparticles

Polymer (50 mg) including PMMA, PLLA, and PLA was dissolved separately in dichloromethane (10 mL) before celecoxib (5 mg) was added to form a homogeneous organic phase. The organic phase was added into 40 ml 2% PVA solution, followed by homogenization (ATS Engineering Inc, Shanghai, China) at 800 bar for 3 cycles. The suspension was added into 150 ml 2% PVA solution, after a continuous stirring for 4 hours to evaporate dichloromethane, the suspension was centrifuged at 13,000 rpm for 25 minutes at 4°C. The pellet was resuspended in de-ionised water by sonication for 60 s and centrifuge again, repeated the wash steps twice. After washes, the pellet was resuspended in de-ionised water by sonication and maintained at −80°C for 2 hours before being lyophilized for 37 hours.

### Drug encapsulation efficiency (EE) and drug loading

Percentage of celecoxib embedded in the prepared nanoparticles was quantified by Ultra-High-Performance Liquid Chromatography (UHPLC) (Accela, Thermo Scientific Inc., San Jose, USA) at 254 nm wavelength. The prepared celecoxib-loaded nanoparticles (50 mg) were dissolved in dichloromethane (10 mL), followed by sonication for 30 min. The resulting supernatant (1 mL) was left under fume hood to evaporate, of which the residue was dissolved in a 30/70 v/v water/ formic acid solution (1 mL) under vigorous vortexing for 10 min. The final solution was transferred into a UHPLC vial using a syringe equipped with PVDF membrane filter (0.22 μm). Loading capacity of celecoxib was calculated as below:


$$\text{LP}\%=\frac{Entrapped\;drug\;weight}{Total\;polymer\;weight+entrapped\;drug\;weight}\times \text{100}\%$$ (3)

### Self-assembled monolayer preparation

A self-assembled monolayer of (3-Mercaptopropyl) trimethoxysilane was prepared on gold QCM sensors to replicate the surface chemistry of silicone elastomer used in the friction experiments. The SAM was measured using ellipsometry and contact angle goniometer to ensure its quality (data shown in S1 Table). To further test the properties of the SAM, it was immersed in a 0.5 M HCl solution for 10 minutes, which should hydrolyse the methoxide groups, leaving the surface more hydrophilic.

### Tribological tests

Tribological tests were performed using the previous method [54]. Stainless steel sphere and silicone elastomer were cleaned with ethanol and deionized water for 10 minutes, and subsequently treated under UV-ozone for 10 minutes. Different types of nanoparticles were dissolved in PBS (Sigma-Aldrich) containing 0.1% HA and 0.5% SDS under stirring condition until the particles were evenly dispersed. Tribological characteristics of the suspension containing nanoparticles (12.5 mL) were investigated by Mini-Traction Machine (PCS Instrument, UK) at 37°C, as a function of sliding velocity (1−100 mm s^-1^), under a maximum force of 7 N. Interfacial friction was measured between a stainless steel sphere of a diameter 19.05 mm and a silicone elastomer disc, with each test carried out six times.

### Surface adsorption of NPs

Quartz Crystal Microbalance (openQCM, Novaetech, Italy) was used to examine the surface adsorption process of nanoparticles, whereby a silane monolayer was prepared on the QCM sensor. Functionalized QCM sensor (resonant frequency of 10 MHz) was exposed to a continuous flow of PBS buffer (flow velocity 0.35 mL/min) to reach equilibrium before the input liquid was switched to 0.1% celecoxib-loaded nanoparticles dissolved in PBS. Once the adsorption reached equilibrium, inlet liquid was changed back to PBS solution in order to remove any physi-sorbed nanoparticles.

### Atomic force microscopy (AFM)

AFM measurements (intermittent contact mode) were carried out in ambient environment with controlled temperature (17°C), using a NanoWizard II (JPK Instruments Ltd, Germany) fitted with silicon cantilevers (Windsor Scientific, UK), of which the nominal spring constant is 42 N m^−1^ and resonance frequency is 320 KHz. The acquired AFM images were analyzed by Gwyddion software and ImageJ 1.52a.

### Drug release kinetics

The release profile of each drug-loaded nanoparticle suspension was examined in a shaker (60 rpm, 37°C) according to drug release kinetics of nanoparticles [70]. The drug loaded nanoparticles were put into a dialysis bag (molecular weight cutoff, 8000–14000), containing 1 mL 0.22% HA, which is the average level of HA in the synovial fluid of non-OA human knee joints. Put the dialysis bag into a 15 mL tube containing 10 mL PBS plus 0.3% SDS (m/v) release medium. Medium (0.5 mL) was taken out and replaced by fresh PBS solution (0.5 mL). The amount of released celecoxib was evaluated by HPLC (Thermo Scientific, America).

### Cytotoxicity of nanoparticles suspensions

A standard Microtubules (MTS) array was used to evaluate the in-vitro cytotoxicity of the NPs formulations on primary human osteoarthritic chondrocytes and fibroblasts [71]. The cells were seeded into 96-well plates at a density of 6,000 cells per well for 24 hours. Cells were then exposed to various formulations and were incubated under 37^o^C and 5% CO_2_ for either one or five days. On the day of the test, 20 μl of the MTS reagent Thaw Cell Titer 96AQ (Promega, USA) was added per well, and the cells were incubated for two hours. Afterwards, their absorbance at 490 nm was examined with a microplate reader (Synergy HT, BioTek Instruments, USA). The number of cells was quantified from the absorbance values based on a reference curve.

## Supporting information

S1 Table Characterisation of Silane SAM after surface treatment. (XLSX)

S1 Fig Raw data for all figures. (XLSX)
